# Development of High-Antifouling PPSU Ultrafiltration Membrane by Using Compound Additives: Preparation, Morphologies, and Filtration Resistant Properties

**DOI:** 10.3390/membranes6020035

**Published:** 2016-06-21

**Authors:** Jie Liu, Zhencheng Zhong, Rui Ma, Weichen Zhang, Jiding Li

**Affiliations:** 1 National Institute of Clean and Low Carbon Energy, Beijing 102209, China; zhongzhencheng@nicenergy.com (Z.Z.); marui@nicenergy.com (R.M.); zhangweichen@nicenergy.com (W.Z.); 2 The State Key Laboratory of Chemical Engineering, Department of Chemical Engineering, Tsinghua University, Beijing 100084, China; lijiding@mail.tsinghua.edu.cn

**Keywords:** polyphenylsulfone, porous asymmetric membrane, compound additives, filtration resistance, properties and characterization

## Abstract

In this study, flat sheet asymmetric polyphenylsulfone (PPSU) ultrafiltration membranes with enhanced antifouling properties were prepared with a non-solvent induced phase separation (NIPS) method through compound additives containing a polymeric pore-forming agent, a small molecular non-solvent and a surfactant. The formation processes of the porous asymmetric membranes with different kinds of additives were studied in detail, and the microstructure controllable preparation of membrane was achieved by establishing a bridge between the membrane preparation parameters and separation performances. All prepared membranes were characterized by using a scanning electron microscope (SEM), contact angle analysis, porosity, maximum pore size, water and BSA solution permeability studies. The performance efficiency of the membrane was evaluated by using BSA as a model foulant in terms of permeability, solute rejection (*R*), *R_m_* (membrane inherent resistance), *R_c_* (cake layer resistance), and *R_p_* (pore plugging resistance). The results showed that when the compound additives were used, the inter-connected pores were observed, maximum pore size, contact angle and membrane filtration resistance decreased, while the porosity increased. When PVP compound additives were added, the water flux increased from 80.4 to 148.1 L/(m^2^·h), the BSA rejection increased from 53.2% to 81.5%. A similar trend was observed for membranes with added PEG compound additives; the water flux and BSA rejection simultaneously increased. The filtration resistance decreased as a result of compound additives. The uniformity of membrane and the number of effective pores could be enhanced by adding compound additives through the cooperation of different additives.

## 1. Introduction

Membrane technology is widely applied in water treatment and has received more attention since it is an outstanding process for the removal of salts, particles, turbidity and organic matter from ground water, industrial and municipal wastewater [1,2,3,4]. In particular, as an efficient low pressure filtration technology, ultrafiltration (UF) membranes have attracted a considerable amount of attention for water clarification and disinfection by size exclusion [5,6,7]. However, with the development of the economy and elevated attention for environmental protection, the performance requirement of the separation membrane is much higher. At present, how to control the membrae preparation conditions to obtain the required membrane structures and performances is the key scientific problem in membrane technology. Membrane fouling is a serious problem in the case of protein separation because hydrophobic protein molecules easily deposit on the membrane surface or plug membrane pores, which tends to suffer a severe decrease in pure water flux during operation [8]. The backwashing and chemical cleaning cannot effectively recover the membrane separation performance, which leads to low water recovery rate and high treatment cost. Thus, membrane fouling caused by dissolved organic matters is a key issue for the successful application of UF membranes.

The literatures have reported that there are three key mechanisms for the contamination of UF membranes by protein molecules [9,10]: (1) membrane pore narrowing caused by irreversible adsorption; (2) solute adsorption and pore blocking; (3) concentration polarization and cake layer formation on the membrane surface. According to the pollution mechanisms of protein molecules, membrane properties including hydrophilicity, electric charge and surface roughness have a great influence on the alleviation of membrane fouling. Water is preferentially adsorbed and arranged orderly on the membrane surface due to the hydrogen bonds effect between hydrophilic membrane surface and water molecules. If hydrophobic proteins intend to permeate through the membrane, they have to overcome the energy barrier to disrupt the orderly arranged water molecules. Thus, increasing the membrane hydrophilicity can effectively minimize membrane fouling. It was reported by Zhao *et al*. [11] that the addition of amphiphilic block copolymers (mPEG-b-PS) can improve the hydrophilicity of PES hollow fiber membranes. The hydrophilic modification was a good method to improve the antifouling properties. Peeva *et al.* [12] used UV initiated grafting method to fabricate low-fouling thin-layer hydrogel composite membranes. The hydrophilic monomer poly(ethylene glycol) methacrylate was grafted onto PES UF membranes, and the antifouling performance improvement by the membrane hydrophilization was significant. The charged membrane can also be used to reduce membrane fouling. In general pH = 7, protein molecules are usually negative charged. As the membrane surface is positive charged, the proteins easily deposit on the membrane surface due to the interaction force between positive and negative electric charge. When the membrane surface has the same electric charge as the proteins, the membrane is not easily polluted. Therefore, the negatively charged membrane material with mutual exclusive force on proteins can effectively prevent the membrane from fouling. Hwang *et al.* [13] found that PPSU/PEI blend membranes possessed a weak negative charge and exhibited good resistance to the negatively charged humic acid due to the effect of electro-static repulsion. Surface roughness is another factor that influences the protein fouling process. On the one hand, membrane surface roughness can increase the possibility of protein molecules adsorbed on the membrane. On the other hand, the relatively high roughness can improve the membrane surface turbulence degree, which prevents the protein molecules from forming cake layer. These two factors result in the effect of roughness on protein fouling on membrane surface. Zhao *et al.* [14] found that PVDF/GO UF membranes had a smoother surface with a higher efficient filtration area, which would enhance antifouling properties. The surface of pure PVDF membrane presented high roughness with several obvious “peaks” and “valleys”. The membrane could be easier fouled with a higher roughness owing to contaminants accumulating in the “valleys” of the rough membrane surface [15].

**Figure 1:**
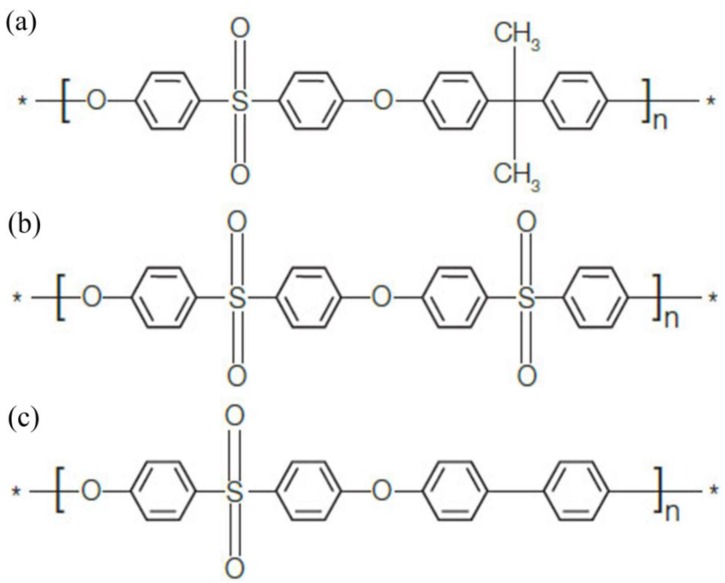
Molecular structures: (**a**) PSf; (**b**) PES; (**c**) PPSU.

To circumvent the above problems, it is necessary to develop a new kind of antifouling membrane material to solve the problem of membrane fouling in the UF process. Polyphenylsulfone (PPSU) is a new member of the family of polysulfone resins. Figure 1 shows the molecular structures of PSf, PES and PPSU. PPSU is a kind of non-crystallizing high performance engineering plastics. It not only combines the advantages of high glass-transition temperature of PES and low water absorption of PSf, but also shows high chemical resistance, outstanding mechanical strength and resistance to several organic compounds [16]. The surface of PPSU resin is negatively charged, which can be used to prepare an excellent separation membrane through the non-solvent induced phase separation (NIPS) method. It was found that the PPSU membrane surface had a negative charge over a pH range of 2 to 10 [13]. However, due to the hydrophobic nature of PPSU, the protein molecules can easily adhere to or accumulate on the membrane surface. PPSU membranes usually suffer severe membrane fouling during water treatment. Furthermore, the asymmetric membrane prepared by NIPS method is prone to form a dense surface layer. Thus, to date, research on preparation and characterization of PPSU UF membranes in water treatment with common pore-forming agents has rarely been discussed in the literature. PPSU membranes are usually used in the fuel cell proton-conducting membrane [17,18,19,20,21], gas separation [22], organic solvent resistant nano-filtration membranes [23,24,25] and pervaporation process [26,27]. The PPSU resin and proteins are both negatively charged. Due to the existed repulsive force between PPSU and proteins, PPSU becomes the superior candidate for the UF membrane to remove proteins.

The dispersion and pore-forming properties of PPSU casting solution may be improved by the compound additives, which is favorable to the formation of membranes with proper pore size distribution and high water flux. In this paper, PPSU UF membranes with enhanced antifouling properties were fabricated by NIPS method, and the effects of single and compound additives on the separation performance of such prepared membranes were studied. Furthermore, the resulting membranes were applied in the protein removal process, and the filtration resistances were analysed with Darcy’s law.

## 2. Experimental Section

### 2.1. Materials

PPSU was purchased from BASF (Ultrason^®^ P 3010 nat, Ludwigshafen, Germany), with *M_w_* = 48,000, *M_w_*/*M_n_* = 2.7. *N*-methyl-2-pyrrolidone (NMP) was obtained from Fuchen Chemical Reagent Factory (Tianjin, China), and it was used as the solvent for PPSU. Polyvinylpyrrolidone (PVP, *M_w_* = 15,000), Tween-80, polyethylene glycol (PEG, *M_w_* = 6000) and 1,2-propylene glycol (PG, 99%) were used as additives, and they were supplied by Guangfu Chemical Industry Research Institute (Tianjin, China). Bovine serum albumin (BSA, *M_w_* = 68,000) was used as a model foulant in permeability experiment. BSA was reagent grade, and purchased from Beijing Jingke Biotechnology Co., Ltd. (Beijing, China). All chemicals were used as received.

### 2.2. Membrane Preparation

During the membrane preparation, the interfacial wettability between the casting solution and coagulation bath, the exchange rate of solvent and non-solvent, and the solidification rate of PPSU may be effectively controlled by adding compound additives. Thus, typical additives including polymeric pore-forming agent (PVP and PEG), small molecular non-solvent (PG) and surfactant (Tween 80) were used to fabricate the PPSU membranes.

A mixture of PPSU, NMP and additives was dissolved in a hermetically sealed glass flask to form homogeneous casting solution, then the casting solution was degassed at 70 °C for 24 h in an oven. After that, the casting solution was directly cast into flat sheet membranes on the glass plate using a finely polished glass rod by maintaining a membrane thickness of 0.20 mm approximately without using the nonwoven support. The flat sheet membranes were solidified by quenching in a water bath at 30 °C for 0.5 h. Finally, the fabricated membranes were stored in freshwater for 48 h to guarantee complete remove of the residual solvent. Flat sheet membranes were dried in ambient air. Table 1 and Table 2 list the preparation parameters and casting solution compositions of PPSU membranes.

### 2.3. Characterization

#### 2.3.1. Scanning Electron Microscope (SEM)

The surface and cross-section morphologies of PPSU membranes were observed by scanning electron microscope (JEOL, JSM-7401F, Tokyo, Japan) using an acceleration voltage of 3.0 kV. For this purpose, the membranes were frozen in liquid nitrogen and then broken into pieces, which were transferred into the microscope chamber with a sample holder after sputtering with gold as the conductive material.

#### 2.3.2. Maximum Pore Size (Bubble Point Method)

PPSU membranes were immersed in alcohol for 15 min before test. The pretreated membranes with an area of 8.0 cm^2^ were installed in the homemade instrument, then N_2_ was blown into one side of the membranes and forced to permeate across the membranes from the topside to the downside through its pores. The pressure at which the first bubble came out was measured as the bubble point pressure. The maximum pore size was obtained using the bubble point method and calculated by [28]:

(1) $$r_{\max}=\frac{C\gamma }{2P}$$

where *r_max_* is the maximum pore size of the membrane (μm). *γ* is the surface tension at the alcohol/air interface, which is equal to 22.32 mN/m at room temperature (25 °C). *P* is the bubble point pressure, Pa. *C* is a constant, 2860 when *P* is in Pa. At least five flat sheet membranes were used for measuring the maximum pore size, and the average values were reported.

#### 2.3.3. Contact Angle Analysis

The contact angle is often used to describe the surface hydrophilicity [29]. In general, membrane hydrophilicity is higher while its contact angle is smaller. All prepared membranes were washed completely in distilled water, and the membrane surface was mopped with tissue paper. Sessile drop method was employed to measure the contact angles of the membranes. The contact angle measurements were carried out with a contact angle meter (OCA15EC Dataphysics, Filderstadt, Germany) at 25 °C. The contact angles were measured within 5 s. The reported values were the averages of the contact angles of five droplets.

#### 2.3.4. Membrane Permeability

The pure water flux of PPSU UF membranes with an effective area of 22.5 cm^2^ were determined by using the cross-flow filtration. The schematic diagram of cross-flow filtration system was described in our previous work [30]. The compaction of each fresh membrane was carried out with deionized water for 20 min at a trans-membrane pressure of 1.5 MPa, which was higher than the operating pressure. The compact membranes were washed thoroughly before used. Pure water was forced to permeate from one side to the other side of PPSU membranes, trans-membrane pressure was set to 0.1 MPa, the test temperature was fixed at 25 °C, and the flux was calculated on the basis of the effective membrane area.

The permeability of BSA was also investigated. The BSA solution was applied to the membrane using the method described above at the same process conditions. The BSA solution with the concentration of 450 mg/L was used. The BSA concentrations in feed and permeate were measured at the wavelength of 280 nm by an ultraviolet visible spectrophotometer (UV2102PCS, Shanghai unique Instrument Co., Ltd., Shanghai, China). 

The separation performance of the PPSU membranes was evaluated on the basis of the flux and rejection. Each data was the average of at least five parallel experiments.

The flux (*J*) at steady state was calculated by the following equation:

(2) $$J=\frac{W}{A\times t}$$

where *W* represents the volume of the collected permeation, L; *A* is the effective membrane area, m^2^; and *t* is the permeation time, h.

The rejection of the BSA was expressed by rejection rate, which was obtained as follows:

(3) $$R=\frac{C_{0}-C_{i}}{C_{0}}$$

where *C*_0_ and *C_i_* represent the BSA concentrations (mg/L) in the feed and permeate respectively.

#### 2.3.5. Porosity

Porosity of the membrane played an important role on permeation and separation. Membrane porosity was measured by the method of dry-wet weight. In order to determine membrane porosity, membranes were immersed in deionized water for 4 h at 25 °C. The wet membrane from deionized water was weighed after its surface water was wiped by tissue paper. The wet membrane was dried in a vacuum oven at 60 °C for 24 h before it was weighed [31]. From the two weights (wet sample weight and dry sample weight), the overall porosities (*ε*) of the membranes were calculated according to the equation [32]:

(4) $$\varepsilon =\frac{\left(W_{w}-W_{d}\right)/\rho_{H_{2}O}}{\left(W_{w}-W_{d}\right)/\rho_{H_{2}O}+W_{d}/\rho_{p}}\times 100\%$$

where *W_w_* and *W_d_* are the wet and dry weight of the membranes, respectively, *ρ_H__2__O_* = 1.0 g/cm^3^, *ρ_p_* = 1.29 g/cm^3^. At least ten flat sheet membranes were measured.

#### 2.3.6. Filtration Resistance Analysis

All filtration experiments were carried out in a cross-flow UF system. Membrane fouling can be quantified by the different kinds of resistances appearing during the filtration [33]. The fouling resistance was mainly owing to the formation of a cake layer on the membrane surface and membrane pore plugging.

The antifouling properties of the PPSU membranes were characterized according to the literature [34]. The pure water flux of the PPSU membrane (*J_o_*, L/(m^2^·h)) was measured at 0.1 MPa. BSA was used as the model protein to study the antifouling properties of the membranes. The 450 mg/L BSA solution was prepared and filtered through the membrane for 60 min. *J_t_* (L/(m^2^·h)) is the steady-state flux of BSA solution. Then the membranes were flushed with pure water for 20 min and then again the pure water flux (*J_i_*, L/(m^2^·h)) was measured.

According to Darcy’s law [35]:

(5) $$J=\frac{\Delta P}{\mu R_{t}}$$

where *J* is membrane flux, L/(m^2^·h); Δ*P* is trans-membrane pressure, MPa; *μ* is viscosity of feed water (25 °C), Pa·s; *R_t_* is total resistance, m^−1^.

*R_t_* included membrane inherent resistance (*R_m_*), cake layer resistance (*R_c_*) and pore plugging resistance (*R_p_*), m^−1^. They were calculated according to the equations:

(6) $$R_{t}=R_{m}+R_{c}+R_{p}$$

(7) $$R_{t}=\frac{\Delta P}{\mu J_{t}}$$

(8) $$R_{m}=\frac{\Delta P}{\mu J_{o}}$$

(9) $$R_{p}=\frac{\Delta P}{\mu J_{i}}-R_{m}$$

(10) $$R_{c}=R_{t}-R_{p}-R_{m}$$

where *J_o_* is the initial flux of pure water, L/(m^2^·h); *J_t_* is the steady-state flux of BSA solution, L/(m^2^·h); *J_i_* is the steady-state flux of pure water of membranes used 60 min in BSA solution after cleaning the cake layers, L/(m^2^·h).

## 3. Results and Discussion

### 3.1. Morphological Studies

**Figure 2:**
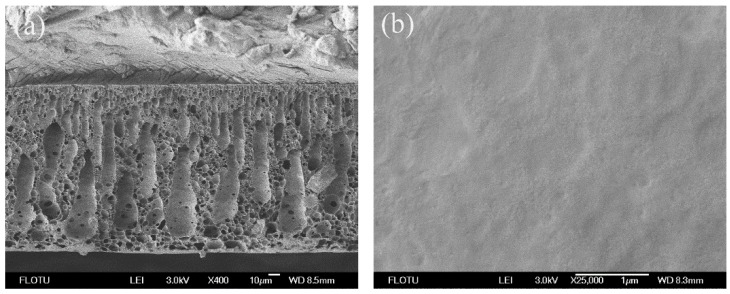
SEM photomicrographs of neat PPSU membranes: (**a**) cross-section; (**b**) top surface.

Figure 2 shows the SEM photomicrographs of neat PPSU membrane. The finger-like and tear drop shape structures were observed in the cross-section. The top surface of the neat PPSU membrane was flat and dense with no appreciable pores. The neat PPSU membrane showed almost no water flux at the test pressure (0.1 MPa). This may be due to the hydrophobic nature of neat PPSU resulting in a very dense layer on the membrane top surface attenuating the membrane permeability.

**Figure 3:**
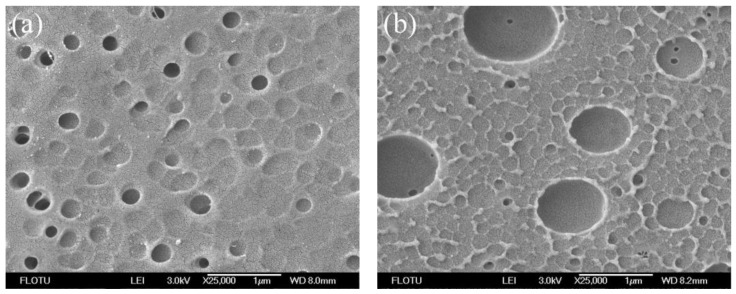

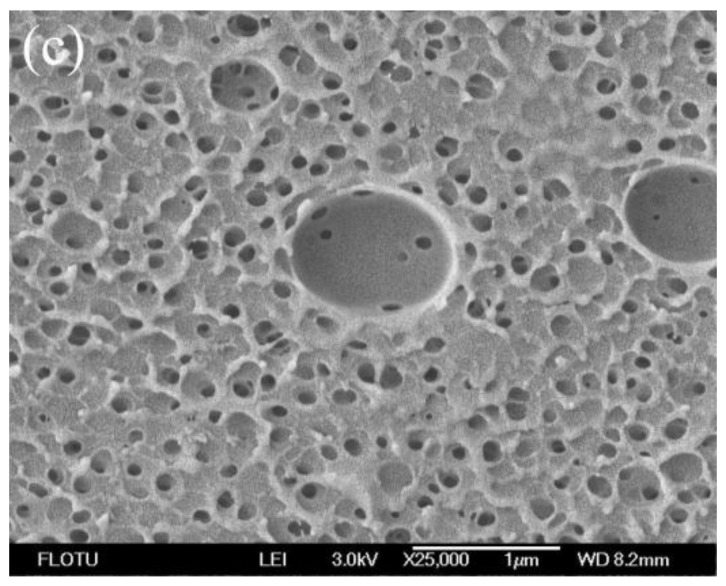
SEM photomicrographs of the top surface of PPSU UF membranes prepared by single and compound additives with PVP as a polymeric pore-forming agent: (**a**) PPSU2; (**b**) PPSU3; (**c**) PPSU4.

**Figure 4:**
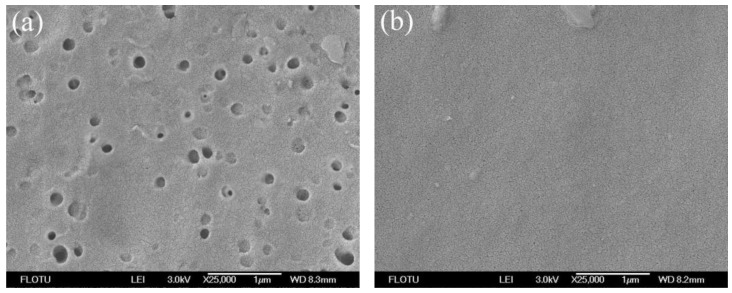
SEM photomicrographs of the top surface of PPSU UF membranes prepared by single and compound additives with PEG as polymeric pore-forming agent: (**a**) PPSU5; (**b**) PPSU6.

**Figure 5:**
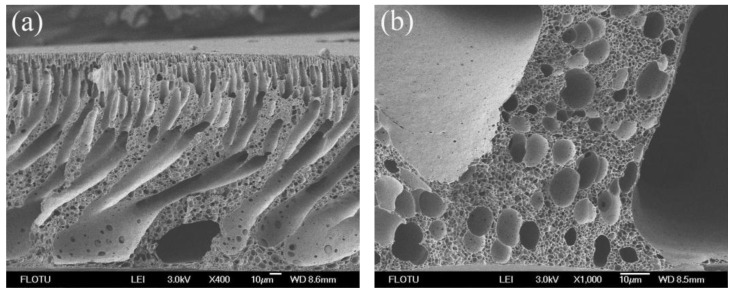

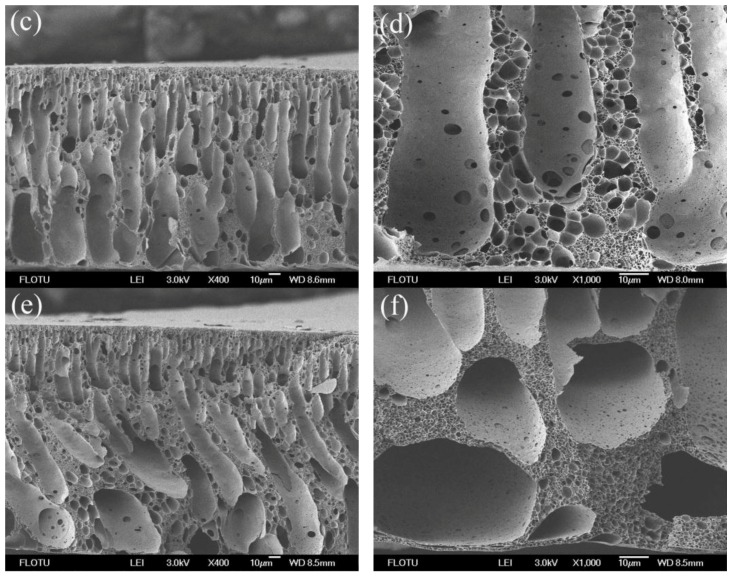
SEM photomicrographs of cross-section of PPSU UF membranes prepared by single and compound additives with PVP as a polymeric pore-forming agent: (**a**,**b**) PPSU2; (**c**,**d**) PPSU3; (**e**,**f**) PPSU4.

**Figure 6:**
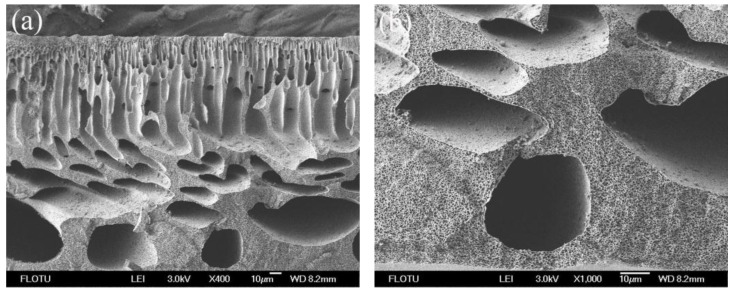

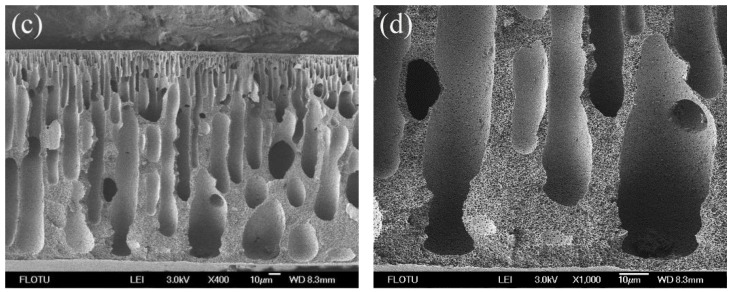
SEM photomicrographs of cross-section of PPSU UF membranes prepared by single and compound additives with PEG as polymeric pore-forming agent: (**a**,**b**) PPSU5; (**c**,**d**) PPSU6.

The surface and cross-section structures of flat sheet PPSU membranes are the most critical part, helping to identify the role of the membrane in the mechanism of permeation and rejection. Figure 3, Figure 4, Figure 5 and Figure 6 show the SEM photomicrographs of PPSU membranes prepared by single and compound additives with PVP and PEG as polymeric pore-forming agents. All the membranes were found to have asymmetric structure (typical structure of UF membranes) consisting of a dense top layer (air side), a porous sublayer and a small portion of sponge-like bottom surface layer (glass side) as seen from SEM photographs. The skin layer acted as a separation layer and the support layer provided the mechanical strength. The sublayer seemed to have finger-like cavities as well as macrovoid structures.

In Figure 5a and Figure 6a, the cross-sections of PPSU membranes prepared by single pore-forming agent PPSU2 (PPSU with 10 wt % PVP) and PPSU5 (PPSU with 10 wt % PEG) tended to form macrovoid structure near the bottom surface layer, and the pore number on the wall of macrovoid structure was small (Figure 5b and Figure 6b). Meanwhile, the pore size distribution on the top surface of PPSU2 and PPSU5 was not uniform (Figure 3a and Figure 4a). Due to the high mutual affinity of NMP for water and addition of polymer pore-forming agent (PVP and PEG), instantaneous demixing resulted, leading to the formation of macrovoid structure in the sublayer of the prepared membranes [36]. When the compound additives containing polymeric pore-forming agent, small molecular non-solvent and surfactant were used for PPSU3 and PPSU6, uniform structures were observed both in the cross-section and top surface of membranes. The connectivity level of pores was greatly improved, and the pore number on the wall of macrovoid structures increased a lot (Figure 5d and Figure 6d). Meanwhile, there appeared more effective pores on the top surface of PPSU membranes, which became small and uniform (Figure 3b and Figure 4b). The addition of compound additives caused significant suppression of the macrovoid structure in the bottom layer (Figure 5c and Figure 6c), which meant the compound additives had substantial role on the precipitation rate [37]. The big macrovoid size could be suppressed by adding the second additive Tween 80 for PPSU3 and PPSU6. As a pore former, the pore formation capability of Tween 80 was not as good as PEG and PVP. When compared with PPSU3 and PPSU4, with the increase in the concentration of compound additives, the pore size both in the top surface and cross-section increased for PPSU4 (Figure 3c and Figure 5e). The results demonstrated that the membrane structures could be adjusted and optimized by compound additives.

### 3.2. Structural Parameters and Properties of the Membranes

Table 3 lists the structural parameters and separation performances of the PPSU UF membranes with single and compound additives. Compared with single additives (PPSU2 and PPSU5), the compound additives (PPSU3 and PPSU6) with the same additive dosage (10 wt %) could exponentially increase both the permeation flux and BSA rejection of PPSU membranes. The water flux increased from 80.4 to 148.1 L/(m^2^·h) for PVP compound additives, and from 74.3 to 129.5 L/(m^2^·h) for PEG compound additives. The BSA rejection increased from 53.2% to 81.5% for PVP compound additives, and from 62.3% to 89.5% for PEG compound additives. Meanwhile, the maximum pore size and contact angle of separation membranes decreased slightly, and the porosity increased significantly. The porosities and hydrophilicity of PPSU3 and PPSU6 were much higher in comparison to PPSU2 and PPSU5. Meanwhile, there appeared more effective pores on the top surface (separation layer) of PPSU3 and PPSU6 membranes, which became small and uniform. Consequently, the mebranes (PPSU3 and PPSU6) had both high pure water flux and BSA rejection. The results demonstrated that the optimization of separation layer and cross-section structure of the membrane could overcome the trade-off phenomenon. When the dosage of compound additives increased from 10 to 15 wt % (from PPSU3 to PPSU4), the maximum pore size, porosity and permeation flux increased, the contact angle and BSA rejection decreased. Due to the cooperation of different additives, the number of effective pore increased greatly, and the PPSU membranes with uniform and optimized structures were obtained. Furthermore, the porous membranes with different maximum pore sizes, permeation flux and BSA rejections could be easily obtained by the addition of different kinds and amounts of additives (PPSU3 and PPSU6).

The composition of casting solution had a great influence on the structure and performance of the PPSU membranes. In order to improve the structural stability and hydrophilicity of PPSU membranes, the pore-forming properties of the casting solution were elevated by the cooperation of different kinds of additives.

The polymeric pore-forming agent (like PVP and PEG) mainly played the role of dispersion and thickening. This was because the total polymer concentration increased with polymeric pore-forming agent addition, so the solvent had to accommodate more macromolecules, leading to less thermodynamic stability [38]. The addition of polymeric pore-forming agent could promote the formation of macrovoids in the membrane. Thus, it seemed that it was necessary to adopt another additive as a pore former to adjust the membrane structure. The permeation and emulsification of the surfactant (Tween 80) were very strong. Tween 80 could decrease the interfacial surface energy of the polymer solution, and thus increased the affinity between NMP and water. This function enhanced the exchange rate between NMP and water [39]. On the other hand, hydrogen bond could be formed between Tween 80 and NMP, which decreased the solubility of NMP. Thus, the viscosity of the polymer solution increased. It had been reported that the increase in viscosity of the casting solution resulted in a longer phase separation time [39,40]. Meanwhile, the leakage rate of Tween 80 from the polymer solution was slow due to steric hindrance [30]. Consequently, the phase separation delay time became longer [30]. Thus, it was likely that the kinetic effect of Tween 80 could partially offset the impact of polymeric pore-forming agent addition, leading to macrovoid size reduction. As for low molecular additive, PG was a weak non-solvent for the casting solution. PG could easily diffuse out during the membrane formation. Its presence in dope system could produce membranes with good interconnectivity and porosity. Shi *et al.* [41] reported that the addition of micromolecule-alcohol into the polymer dope solution made the resultant membranes exhibit a narrow pore size distribution compared to the effect of polymeric pore-forming agent addition. However, the excessive addition of the low molecular non-solvent led to the instability of the casting solution. Consequently, the addition of compound additives could adjust the exchange rate of solvent and non-solvent during phase inversion process, and optimize the morphology of prepared membranes. To clearly understand the detailed mechanisms of such behavior presented in the membrane fabrication process, further investigation is still needed.

### 3.3. Analysis of Filtration Resistance

Fouling resulted in declined flux and reduced membrane life time. In this study, BSA filtration was carried out by prepared membranes to evaluate the fouling resistant properties. Table 4 lists the values of membrane inherent resistance (*R_m_*), pore plugging resistance (*R_p_*), cake layer resistance (*R_c_*) and total resistance (*R_t_*). As illustrated in Table 4, the membranes PPSU3, PPSU4, and PPSU6 had a lower *R_m_* value, which indicated that highly hydrophilic membrane surface provided lower resistance for water molecules to pass across the membrane [42]. The more hydrophilic membrane surface tended to lower the adhesion for BSA molecules, during operation. As a result, the membranes (PPSU3 and PPSU6) had lower values of *R_c_* and *R_p_* than membranes with a single additive (PPSU2 and PPSU5). All membrane filtration resistances could be reduced by the addition of compound additives. Meanwhile, with the increase in the dosage of compound additives (from PPSU3 to PPSU4), the filtration resistance decreased. The observed changes in the filtration resistance were owing to the improved hydrophilicity and optimized membrane structure as discussed above.

## 4. Conclusions

Flat sheet PPSU membranes were prepared from casting solution containing polymeric pore-forming agent (PVP and PEG), small molecular non-solvent (PG) and surfactant (Tween-80) using the NIPS method. Adding compound additives was an effective method to adjust and optimize the membrane structure and improve the separation efficiency of PPSU membranes. As the compound additives were added, uniform structures were observed both in the cross-section and top surface. The pore permeability was greatly improved, and the pore number on the top surface as well as wall of macro-void structure in the sublayer increased greatly. The pores on the top surface tended to be small and uniform. When PVP compound additives were utilized, the water flux increased from 80.4 to 148.1 L/(m^2^·h), the BSA rejection increased from 53.2% to 81.5%. A similar trend was found for membranes added PEG compound additives. Meanwhile, when the compound additives with the same dosage were used, the maximum pore size and contact angle of separation membranes decreased slightly, the porosity increased obviously. As the concentration of compound additives increased from 10 to 15 wt %, the maximum pore size, porosity and permeation flux increased, the contact angle and BSA rejection decreased. The filtration resistance of the membrane could be reduced by using compound additives, and with increase in the dosage of additive increases, the filtration resistance decreases.

## Figures and Tables

**Table 1:** Preparation conditions of PPSU membranes.

Preparation Conditions	Parameters
Solution temperature (°C)	70
Coagulation bath	pure water
Coagulant temperature (°C)	30
Ambient temperature (°C)	25
Air humidity (%)	40–50

**Table 2:** Casting solution compositions of PPSU membranes.

Membrane Samples	Casting Solution Compositions
PPSU1	PPSU:NMP = 15:85
PPSU2	PPSU:PVP:NMP = 15:10:75
PPSU3	PPSU:PVP:Tween-80:PG:NMP = 15:6:3:1:75
PPSU4	PPSU:PVP:Tween-80:PG:NMP = 15:9:4.5:1.5:70
PPSU5	PPSU:PEG:NMP = 15:10:75
PPSU6	PPSU:PEG:Tween-80:PG:NMP = 15:6:3:1:75

**Table 3:** Structural parameters and separation performances of PPSU UF membranes.

Samples	Maximum Pore Size (μm)	Porosity (%)	Contact Angle (°)	*J_o_* (L/m^2^·h)	*J_t_* (L/m^2^·h)	*J_i_* (L/m^2^·h)	BSA Rejection (%)
PPSU2	0.31 ± 0.02	51.2 ± 3.1	65.1 ± 1.0	80.4 ± 3.3	31.3 ± 1.6	48.3 ± 1.0	53.2 ± 1.5
PPSU3	0.21 ± 0.01	71.5 ± 2.1	63.0 ± 1.2	148.1 ± 2.3	52.3 ± 1.1	90.6 ± 0.9	81.5 ± 1.7
PPSU4	0.26 ± 0.03	80.6 ± 4.5	54.4 ± 2.0	183.4 ± 1.4	63.6 ± 1.4	117.1 ± 1.9	70.1 ± 3.3
PPSU5	0.29 ± 0.02	50.2 ± 3.4	67.3 ± 1.5	74.3 ± 2.4	29.3 ± 1.9	45.7 ± 1.3	62.3 ± 2.4
PPSU6	0.18 ± 0.01	67.9 ± 2.4	60.5 ± 2.3	129.5 ± 1.7	43.9 ± 1.5	75.4 ± 0.8	89.5 ± 1.7

**Table 4:** Analysis of filtration resistances of PPSU UF membranes.

Samples	*R_m_* × 10^11^ (m^−1^)	*R_p_* × 10^11^ (m^−1^)	*R_c_* × 10^11^ (m^−1^)	*R_t_* × 10^11^ (m^−1^)
PPSU2	2.00 ± 0.31	1.34 ± 0.10	0.84 ± 0.15	4.18 ± 0.76
PPSU3	1.08 ± 0.23	0.70 ± 0.19	0.72 ± 0.14	2.50 ± 0.53
PPSU4	0.88 ± 0.13	0.50 ± 0.12	0.68 ± 0.11	2.06 ± 0.41
PPSU5	2.17 ± 0.27	1.36 ± 0.34	0.94 ± 0.19	4.47 ± 0.63
PPSU6	1.24 ± 0.15	0.90 ± 0.22	0.84 ± 0.17	2.98 ± 0.35
